# Alveolar rhabdomyosarcoma confined to the bone marrow with no identifiable primary tumour using FDG-PET/CT

**DOI:** 10.1186/s13569-015-0039-6

**Published:** 2015-11-19

**Authors:** Panagiotis Karagiannis, Nina Guth, Gabriela B. Thoennissen, Christina Bern, Jan Sperveslage, Ilske Oschlies, Carsten Bokemeyer, Wolfram Klapper, Eva Wardelmann, Nils H. Thoennissen

**Affiliations:** Department of Oncology, Haematology and Stem Cell transplantation, University Hospital of Hamburg Eppendorf, Hamburg, Germany; NIHR Biomedical Research Centre at Guy’s and St. Thomas’s Hospitals and King’s College London, London, United Kingdom; Department of Radiology and Nuclear Medicine, University Hospital of Hamburg Eppendorf, Hamburg, Germany; Gerhard-Domagk-Institute of Pathology, University Hospital of Muenster, Muenster, Germany; Institute of Pathology, University Hospital of Schleswig- Holstein, Kiel, Germany

**Keywords:** Rhabdomyosarcoma, Bone marrow, Topotecan/cyclophosphamide, Vinorelbine, Alveolar rhabdomyosarcoma, FDG-PET

## Abstract

**Background:**

Rhabdomyosarcoma (RMS), a malignant tumour of mesenchymal origin which can occur at various sites in the body, is one of the most common soft tissue sarcomas in both children and adolescents, but is rare in adults with a prevalence of less than 1 %. The alveolar subtype of rhabdomyosarcoma (ARMS) is typically characterized by a specific reciprocal chromosomal translocation involving the PAX3 and FKHR or PAX7 and FKHR genes, respectively. ARMS is most frequently seen in childhood, and typically affects the sinuses and soft tissue of the extremities, with approximately 23 % exhibiting metastasis to the marrow. Non-invasive F-18-fluorodeoxyglucose positron-emission tomography (FDG-PET) scans have a high ability to detect lymph nodes, bone, and bone marrow involvement in patients with metastatic RMS, often with higher sensitivity and specificity compared with conventional modalities.

**Case presentation:**

Here, we report an unusual case of ARMS confined to the bone marrow in an older adult that lacked an identifiable primary tumour using FDG-PET/CT and mimicked a haematological disease with pancytopenia but without abnormal findings by FDG-PET/CT. The patient was initially treated with topotecan/cyclophosphamide and subsequently switched to vinorelbine. Due to severe toxicity the treatment was discontinued, however after 7-months follow-up, the patient is still alive with an improved general state of health and only a mild pancytopenia with no need for blood transfusions.

**Conclusion:**

Rhabdomyosarcoma can be limited to the bone marrow with no identifiable primary tumour. This case shows that the use of a bone marrow biopsy in suspected malignancies affecting the bone marrow is irreplaceable.

## Background

Rhabdomyosarcoma (RMS), a malignant tumour of mesenchymal origin which can occur at various sites in the body, is one of the most common soft tissue sarcomas in both children and adolescents, but is rare in adults with a prevalence of less than 1 % [1]. The alveolar subtype of rhabdomyosarcoma (ARMS) is typically characterized by a specific reciprocal chromosomal translocation involving the PAX3 and FKHR or PAX7 and FKHR genes, respectively [2]. ARMS is most frequently seen in childhood, and typically affects the sinuses and soft tissue of the extremities, with approximately 23 % exhibiting metastasis to the marrow [2, 3]. Non-invasive F-18-fluorodeoxyglucose positron-emission tomography (FDG-PET) scans have a high ability to detect lymph nodes, bone, and bone marrow involvement in patients with metastatic RMS, often with higher sensitivity and specificity compared with conventional modalities [4–6]. Here, we report an unusual case of ARMS confined to the bone marrow in an older adult that lacked an identifiable primary tumour using FDG-PET/CT and mimicked a haematological disease with pancytopenia but without abnormal findings by FDG-PET/CT.

## Case presentation

**Fig. 1:**
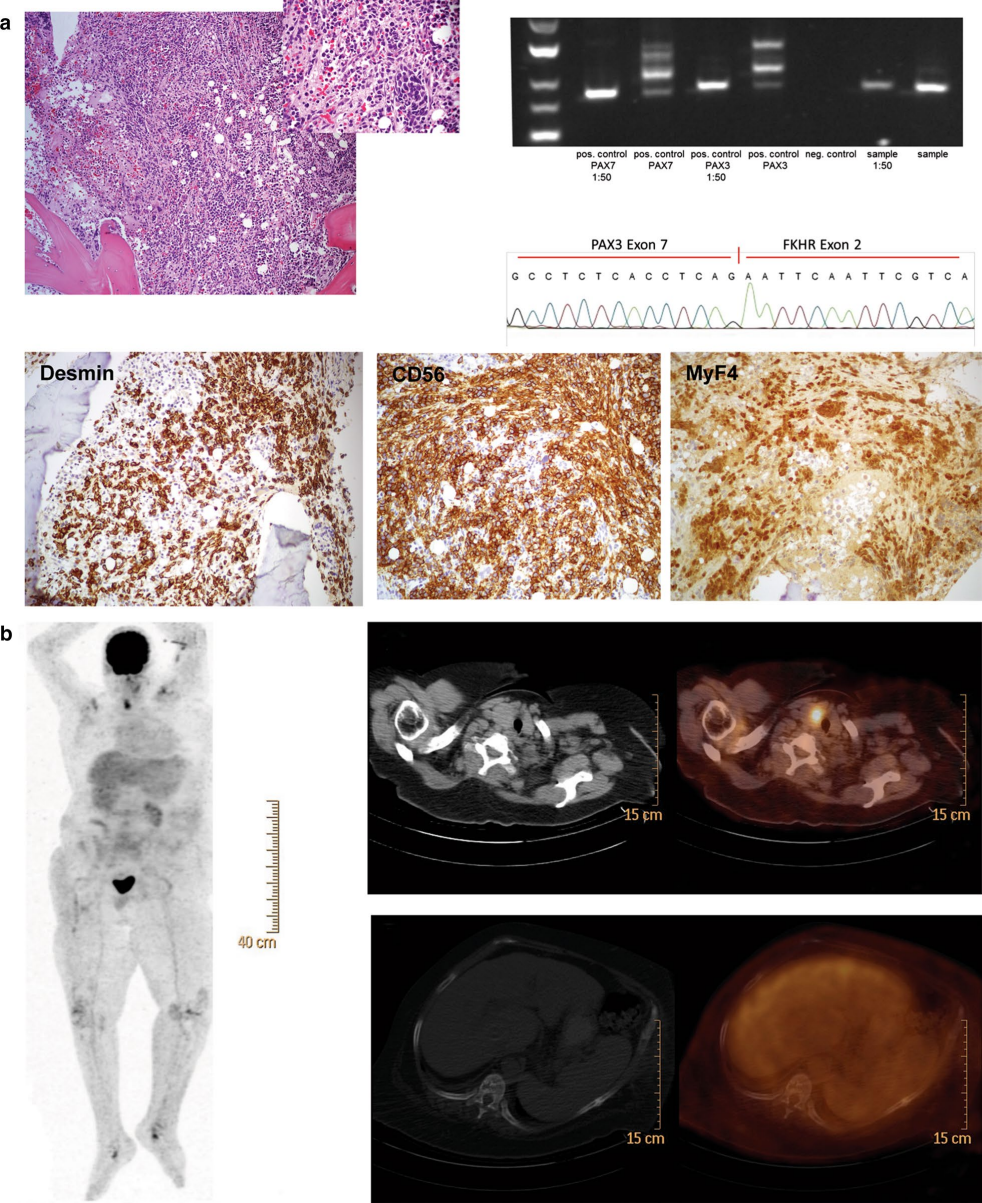
**a** Histological and genetic characteristics of bone marrow infiltrating cells. *Top left* Infiltration of the bone marrow by medium to large atypical cells (×40),* right top corner* (×100); *top right* Gelelectrophoresis of PAX/FKHR RT-PCR including positive and negative controls for PAX7- und PAX3-FKHR fusions in two dilutions (neat and 1:50), below results of Sanger sequencing; *bottom panel* the atypical cells express desmin, CD56 and MyF4. **b** *Left panel* Whole body positron emission tomography (PET-CT); *top right panel* PET-CT indicating one single increased uptake in the right thyroid gland revealing follicular neoplasia; *bottom right panel* haemangioma in the 9th thoracic vertebrae

A 61-year-old Caucasian female presented to an external hospital with a 2 month history of shortness of breath, anorexia and fatigue. She had pre-diagnosed obesity, liver cirrhosis (non-alcoholic fatty liver disease; Child-Pugh A), severe osteoporosis and peripheral edema due to chronic heart disease. On admission, the patient had no fever and no signs of infection, bleeding, haemolysis or acute decompensated heart failure. First laboratory assessments revealed a pancytopenia grade 2–3 including microcytic anaemia with haemoglobin of 7.6 g/dl, white blood cells of 1.7 billion/l and platelets of 74 billion/l, as well as reduced levels of iron, ferritin and vitamin B_12_. The differential leukocyte count revealed a slight left shift, but absence of blasts. Furthermore, electrolytes, lactatdehydrogenase (LDH), and serum markers for liver and kidney function were in normal ranges. Initial symptomatic treatment management mainly included blood transfusions leading to a fast recovery of the patient. To rule out a suspected haematological disease a bone marrow aspiration was performed, which demonstrated no evidence of malignancy. However, since adequate supplementation of iron and vitamin B_12_ did not improve the pancytopenia, and other medical reasons known to cause pancytopenia were excluded (e.g. medications, infections) bone marrow aspiration was repeated, this time including a biopsy. The bone marrow aspiration showed no sign of malignancy. Surprisingly, the immunohistological assessment revealed an infiltration (up to 35 %) of the bone marrow with ARMS (Fig. 1a top panel left) with strong positivity for desmin, CD56 and MyF4 (Fig. 1a bottom panel) and negativity in particular for NGFR, TLE1, Melan-A, MITF, HMB45, and S100 (data not shown). In addition, genetic analysis of the biopsy by reverse transcriptase-polymerase chain reaction (Fig. 1a top panel right) and Sanger sequencing (Fig. 1a top panel right) detected the PAX3-FKHR gene fusion. The patient was immediately referred to our university clinic for further evaluation.

Staging and work up of the occult primary tumour via whole body FDG-PET (Fig. 1b panel left) showed a single site of increased uptake in the right thyroid gland with absence of a corresponding lesion in the integrated computerised tomography (Fig. 1b top panel right). Histological investigation by fine needle aspiration of the respective thyroid gland revealed follicular neoplasia. Furthermore, adenoma of the right adrenal gland and haemangiomas in the 9th and 12th thoracic vertebrae (Fig. 1b bottom panel right) were diagnosed via FDG-PET/CT and were confirmed by magnetic resonance imaging (MRI). Otherwise, whole body FDG-PET/CT and MRI of the spine and head showed no suspicious uptake and/or lesions. In addition, upper gastrointestinal endoscopy and colonoscopy were performed without pathological findings. In summary, diagnosis of an ARMS confined to the bone marrow with no identifiable primary by FDG-PET/CT and which caused insufficient haematopoiesis with the need for regular blood transfusions was made.

Due to co-morbidities and a reduced Eastern Cooperative Oncology Group (ECOG) performance status of three, we started the patient on topotecan 0.75 mg/m^2^/day (days 1–5) and cyclophosphamide 250 mg/m^2^/day (days 1–5) intravenously (i.v.). Chemotherapy-induced side effects included increased anorexia, fatigue with ECOG 4, and haematological toxicity with pancytopenia Common Terminology Criteria for Adverse Events (CTCAE) grade 3–4. Temporary, increased rates of transfusion (red blood cells and platelets) were necessary, as well as the application of iv. antibiotics and granulocyte-colony stimulating factor (G-CSF) due to recurrent fever-in-neutropenia. After the third cycle of chemotherapy, bone marrow aspiration and biopsy were repeated showing maturing trilineage haematopoiesis with no signs of RMS tumour cells. Due to the significant level of high-grade toxicities related to the applied combination chemotherapy and the significant response already achieved we decided to apply monotherapeutic vinorelbine 30 mg/m^2^/d d1 + d8 i.v. (max 60 mg abs./d; q3w) as a consolidation therapy. After the first cycle of vinorelbine, the patient developed muscle and joint pain, nausea/emesis, as well as autonomic neuropathy causing severe constipation. Subsequently, the acute side effects were treated adequately, and follow-up care was introduced.

At present, after a 7-months follow-up, the patient is still alive with an improved general state of health and only a mild pancytopenia with no need for blood transfusions.

## Conclusion

Rhabdomyosarcoma is an aggressive type of sarcoma arising in the soft tissues of the body, like muscles, tendons, and connective tissues [1, 3]. A multimodal treatment including multiagent chemotherapy, radiotherapy, and surgery, is standard of care for this disease, and can lead to a relatively high rate of cure in young patients with local or regional RMS [1, 3, 7]. Improvements in risk stratifications have allowed the treatment assignment of pediatric patients in different therapeutic trials, leading to an increase of up to 70 % in 5 year survival [1, 3, 7]. However, for adult patients, in great part due to rarity of the disease and the lack of clear guidelines for standard treatment, as well as increased prevalence of advanced presentations, clinical outcome is still dismal. The presence of metastases is one of the most adverse prognostic factor in RMS, and bone marrow is a frequent site of tumour dissemination, especially in ARMS [8]. Therefore, with FDG-PET/CT the treatment algorithm has improved through more accurate staging including greater precision in detecting lymph node disease and distant metastases compared with anatomic imaging alone [4–6]. Moreover this case illustrates that frequently bone marrow smears alone are not sufficient to diagnose tumour infiltrations of the bone marrow and therefore a trephine is necessary in any unexplained pancytopenia and will not be replaced by bone marrow aspiration/cytology alone. Furthermore recent emerging data show that new techniques such as whole body diffusion weight (DW)-MRI alongside with molecular and genetic analysis could lead the way to further guide clinical decisions [9].

To the best of our knowledge, we present the first case of an adult patient with ARMS limited to the bone marrow with no identifiable primary tumour by FDG-PET/CT scan. We report this case for its rarity, occurrence in an adult with complete absence of a primary of ARMS, and to increase awareness in the diagnostic, as well as in the staging evaluation for the irreplaceable use of a bone marrow biopsy in suspected malignancies affecting the bone marrow.

## Consent

Written informed consent was obtained from the patient for publication of this case report and any accompanying images. A copy of the written consent is available for review by the Editor of this journal.

## Abbreviations

ARMS: alveolar subtype of rhabdomyosarcoma
CTCAE: Common Terminology Criteria for Adverse Events
ECOG: Eastern Cooperative Oncology Group
FDG: PET- F-18-fluorodeoxyglucose positron-emission tomography
G-CSF: granulocyte-colony stimulating factor
LDH: lactatdehydrogenase
MRI: magnetic resonance imaging
RMS: rhabdomyosarcoma
